# Bio-Inspired Protein-Based Nanoformulations for Cancer Theranostics

**DOI:** 10.3389/fphar.2018.00421

**Published:** 2018-04-27

**Authors:** Yi Gou, Dandan Miao, Min Zhou, Lijuan Wang, Hongyu Zhou, Gaoxing Su

**Affiliations:** ^1^Jiangsu Province Key Laboratory of Inflammation and Molecular Drug Targets, School of Pharmacy, Nantong University, Nantong, China; ^2^Guangzhou Key Laboratory of Environmental Exposure and Health and Guangdong Key Laboratory of Environmental Pollution and Health, School of Environment, Jinan University, Guangzhou, China

**Keywords:** protein nanoparticles, cancer therapeutics, theranostics, drug delivery, cancer diagnostics

## Abstract

Over the past decade, more interests have been aroused in engineering protein-based nanoformulations for cancer treatment. This excitement originates from the success of FDA approved Abraxane (Albumin-based paclitaxel nanoparticles) in 2005. The new generation of biocompatible endogenous protein-based nanoformulations is currently constructed through delivering cancer therapeutic and diagnostic agents simultaneously, as named potential theranostics. Protein nanoformulations are commonly incorporated with dyes, contrast agents, drug payloads or inorganic nanoclusters, serving as imaging-guided combinatorial cancer therapeutics. Employing the nature identity of proteins, the theranostics, escape the clearance by reticuloendothelial cells and have a long blood circulation time. The nanoscale sizet allows them to be penetrated deeply into tumor tissues. In addition, stimuli release and targeted molecules are incorporated to improve the delivery efficiency. The ongoing advancement of protein-based nanoformulations for cancer theranostics in recent 5 years is reviewed in this paper. Fine-designed nanoformulations based on albumin, ferritin, gelatin, and transferrin are highlighted from the literature. Finally, the current challenges are identified in translating protein-based nanoformulations from laboratory to clinical trials.

## Introduction

Cancer, a leading cause of death globally, reaching approximately 8.2 million mortalities yearly, poses an enormous burden on society (Torre et al., 2015; Mehra et al., 2017). Cancer counts as a multifactorial and refractory disease caused by the local tissue micro-environmental and genetic factors intertwined (Hanahan and Weinberg, 2011). Common treatment strategies, including radiotherapy and/or chemotherapy with surgery, result in high treatment failure rate (Aoun et al., 2015; Kouchakzadeh and Abbas Shojaosadati, 2016). The reasons of treatment failure are generally diverse: (1) cancer is commonly detected at a later stage, thus accuracy and susceptibility of diagnosis and monitoring methods for early-stage cancer require further improvement (Ge and Liu, 2013; Torre et al., 2015); (2) in most cases, the conventional chemotherapy have been disappointing in efficacy due to multidrug resistance (MDR) and severe side effects (Gelperina et al., 2005; Pérez-Tomás, 2006; Szakács et al., 2006; Ge and Liu, 2013); (3) the cancer therapeutic biological agents (inclusive of antibodies, proteins and nucleic acids), the new class of anticancer drugs, are commonly unstable in *in vivo* circulation, with rapid degradation and inactivation before reaching the target site (Panyam and Labhasetwar, 2003; Sinha and Trehan, 2003). Accordingly, early detection, effective diagnosis and effective treatment of cancer are needed to be optimized to increase the survival rate and decrease the cancer associated deaths.

“Theranostics,” the portmanteau of therapeutics and diagnostics, has incorporated diagnostic and therapeutic functions into a single nanoplatform (Pene et al., 2009; Chen and Liu, 2016). It is noteworthy that the theranostics have been proposed as a new and revolutionary therapeutic concept in cancer therapy, enabling simultaneous diagnosis and treatment response monitoring using personalized medicine with high accuracy and specificity (Janib et al., 2010; Bardhan et al., 2011). Additionally, it is likely to incorporate numerous different therapeutic drugs into a single theranostics nanoplatform through judicious design to reach synergistic treatment of cancer (Moon et al., 2015; Chen and Liu, 2016). In this regard, theranostics have become a research orientation arousing great concern and been promising in the field of cancer treatment.

Considerable efforts have been recently made to develop new systems for cancer theranostics (Opoku-Damoah et al., 2016; Chen et al., 2017b; Guo et al., 2017; Mohammadi et al., 2017; Tekade and Sun, 2017; Yue et al., 2017). Thus, far, numerous types of theranostic platforms have been reported, consisting of protein nanocarriers (Ng et al., 2011; Chen and Liu, 2016; Truffi et al., 2016), liposome nanocarriers (Wang et al., 2014b; Dai and Yue, 2017), inorganic nanocarriers (Huang et al., 2011; Sharma et al., 2015), polymer nanocarriers (Kamaly et al., 2012; Charron et al., 2015; Sk and Kojima, 2015) and inorganic/organic hybrid nanocarriers (Barreto et al., 2011; Zhu et al., 2015; Li et al., 2017). Among these platforms, protein-based nanoplatforms have aroused the greatest concern by virtue of their biodegradability, biocompatibility, no or low toxicity and ease of modification (Maham et al., 2009; Elzoghby, 2013; Yewale et al., 2013; Chen and Liu, 2016; Kouchakzadeh and Abbas Shojaosadati, 2016). Some proteins, e.g., transferrin and lactoferrin, can be specifically bound to receptor(s) highly expressed in considerable cancer cells via receptor-ligand interaction, enabling the construction of active targeted theranostic nanoplatforms (Kanwar et al., 2016; Wang et al., 2016a, 2017a). Furthermore, in the presence of reactive groups such as –COOH, –NH_2_, and –OH, protein-based theranostic platforms can be further decorated with functional molecules for different purposes. Numerous proteins as versatile platforms for delivering therapeutic agents have been elucidated in previous review articles (Yewale et al., 2013; Paliwal and Palakurthi, 2014; Kouchakzadeh and Abbas Shojaosadati, 2016).

**Figure 1:**
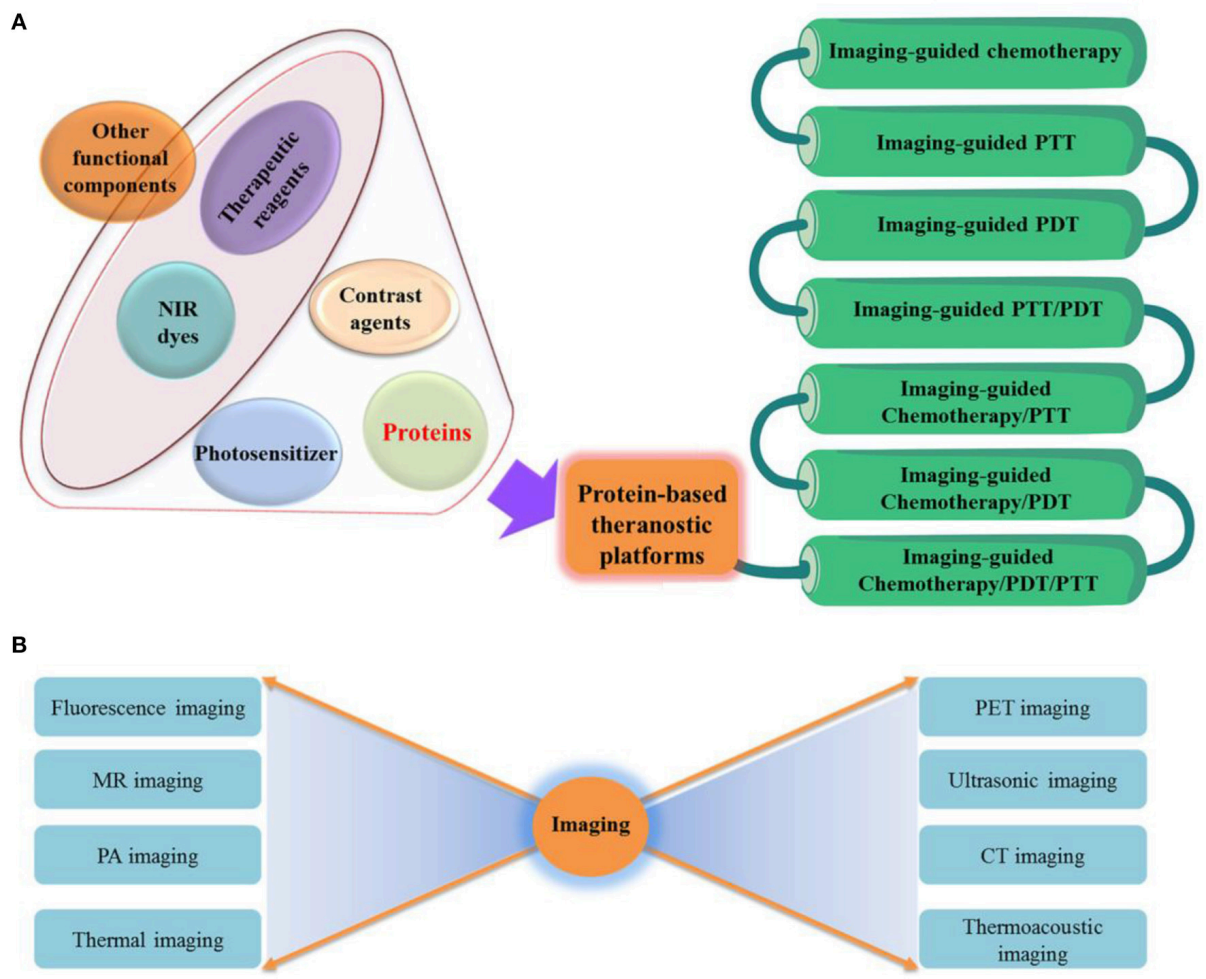
**(A)** Engineering of protein-based nanoformulations as cancer theranostic platforms. **(B)** A series of imaging technologies are incorporated in the theranostic platforms.

This review article stresses the protein-based multifunctional theranostics progress in the past 5 years (Table 1) and summaries critical foundation for further studying theranostics. The therapeutic methods include photothermal therapy (PTT), photodynamic therapy (PDT) and chemotherapy. The diagnostic or imaging methods included magnetic resonance (MR) imaging, fluorescence imaging, computed tomography (CT) imaging, photoacoustic (PA) imaging, positron emission tomography (PET) imaging and so on. The combinatorial strategies between diagnostic and therapeutic methods are summarized in Figure 1. Properties of commonly used proteins, i.e., albumin, ferritin, gelatin, and transferrin, were introduced. The preparation methods and key outcomes of *in vitro* or *in vivo* studies of these protein-based nanoformulations for cancer theranostics were elaborated.

**Table 1:** Overview of the protein-based nanoformulations for cancer theranostics.

**Protein**	**Formulation**	**Application**	**Key outcomes of *in vitro/in vivo* study**	**References**
HSA	HSA-ICG NPs	Fluorescence and PA dual-modal imaging-guided phototherapy	In 4T1 tumor-bearing mice, the normal, tumor and its margin tissue can be clearly identified via fluorescence and PA imaging. After i.v. injection of the NPs followed by imaging-guided precision PDT/PTT, the 4T1 tumor was completely eradicated, no treatments-induced toxicity and tumor recurrence were observed.	Sheng et al., 2014
HSA	HSA-IR825 complex	Imaging-guided PTT	The 4T1 tumor could be clearly identified from 1 to 12 h post injection. After i.v. injection of the complex followed by 808 nm laser irradiation, the 4T1 tumor was significant eradicated and no appreciable toxic side was observed.	Chen et al., 2014c
HSA	HSA-Gd-IR825 nanocomplex	Fluorescence and MR dual-modal imaging-guided PTT	The nanocomplex can be used for effective mapping of the sentinel lymph node nearby tumors, and the mapping signal is clearest at ~30 min post injection. Photothermal ablation of the HSA-Gd-IR825 combined with surgical removal of primary tumors provides significant therapeutic advances in preventing 4T1 tumor metastasis and prolonging animal survival.	Chen et al., 2014a
HSA	HSA–ICG–PTX NPs	Imaging-guided PTT and chemotherapy	The 4T1 tumor could be clearly identified after 4 h post injection. After i.v. injection of the NPs followed by 808 nm laser irradiation, the 4T1 tumor was significant eradicated and no significant toxic side was observed.	Chen et al., 2015a
HSA	HSA-Ce6-PTX-RGD	MR and fluorescence-imaging-guided chemotherapy and PDT	Upon i.v. injection into U87MG tumor-bearing mice, the nano-drug could be effectively tracked by dual modal imaging and shown excellent tumor growth inhibition effect.	Chen et al., 2015b
HSA	Cy5/Qsy21 labeled- Pt(IV)-probe@HSA	Imaging-guided chemotherapy	Upon UV light irradiation, Pt(IV)-probe@HSA showed enhanced cell death and cell apoptosis at both cisplatin-resistant A2780cis and sensitive A2780 cell lines.	Li et al., 2015
HSA	Melanin/PTX-HSA NPs	PA-imaging-guided chemotherapy	*In vitro*, the NPs showed enhanced PA signal and cytotoxicity against MDA-MB-231 cancer cells. *In vivo*, the NPs efficiently accumulated inside the MDA-MB-231 tumor, resulting in inhibiting tumor growth effectively and visualizing tumors photoacoustically.	Sim et al., 2015
HSA	Porphyrin-MB-PTX-HSA NPs	PA and ultrasound-imaging-guided chemotherapy	The MDA-MB-231 tumor and neo-vessels in the tumor region could be clearly visualized after 3 min post injection. Exposure to the focused ultrasound triggered the collapse of the Porphyrin-MB-PTX-HSA NPs, resulting in the PTX-HSA-NPs suppressed MDA-MB-231 tumor growth 10-fold higher than without exposure to ultrasound.	Moon et al., 2015
HSA	HSA@CySCOOH	Fluorescence/PA/thermal multimodality imaging-guided PTT	The 41T tumor can be clearly differentiated from the surrounding normal tissue from 1 to 48 h injection. After injection of the HSA@CySCOOH followed by 808 nm laser irradiation, complete tumor eradication was achieved on 4T1 tumor-bearing mice, with no noticeable toxicity, weight loss, and tumor recurrence being observed.	Rong et al., 2015
HSA	HSA-Ce6 nanoassemblies	Fluorescence/PA/MR triple-modal imaging-guided PDT	The nanoassemblies could be used for PA, MRI and fluorescence triple-modal tumor imaging imaging in 4T1 tumor-bearing mice via i.v. injection. After i.v. injection of the nanoassemblies followed by low-energy NIR irradiation, the 4T1 tumor was completely suppressed without therapy-induced side effects and tumor recurrence.	Hu et al., 2016
HSA	HSA-FePc NPs	PA imaging-guided PTT	The NPs are excellent PA imaging agent, which can clearly show a clear 4T1 tumor microstructure with higher spatial and contrast resolution compared with FePc alone molecules. After i.v. injection, the NPs exhibited efficient cancer therapy, no obviously weight loss and low long-term toxicity were observed.	Jia et al., 2017
HSA	PFT-Hcy-HSA-Cy7-pTFT	Optical and ^19^F MR imaging-guided chemotherapy	The ^19^F signals of PFT-Hcy-HSA-Cy7-pTFT are clearly visible in tumor-bearing mouse. The inhibitory tumor (A549) growth effect of PFT-Hcy-HSA-Cy7-pTFT was found to be 0.8-fold more than that of the pTFT alone.	Lisitskiy et al., 2017
HSA	HSA-gemcitabine/IR780 complex	NIR imaging-guided chemotherapy	Compared to IR780 alone, the complex showed enhanced accumulation and long-term retention in BxPC-3 pancreatic tumor tissues, resulting in inhibiting tumor growth effectively with minimal side effects.	Han et al., 2017
HSA	HSA-coated superparamagnetic iron oxide NPs	MRI and thermoacoustic imaging-guided thermoacoustic therapy	Based on MRI and TA imaging, the NPs provide comprehensive and complementary information for 4T1 tumors. Meanwhile, the NPs mediated TA therapy exhibits excellent anti-tumor efficacy for deep tumor models.	Wen et al., 2017
BSA	BSA functionalized Nano-rGO	PA/ultrasonic dual-modality imaging-guided PTT	The theranostic agent not only showed rapid and significant PA signal enhancement in the MCF-7 tumor area, but also can effectively kill tumor cells *in vivo* with no noticeable organs toxic.	Sheng et al., 2013
BSA	squaraine@BSA	Dual-functional NIR probe for targeted optical imaging and selective PTT of cancer.	The optimal imaging and PTT window for KB xenografted tumor was within 6 h post-injection. After a tail veins injection of the theranostic agent followed by 680 nm laser irradiation, the KB xenografted tumor was significantly suppressed.	Gao et al., 2014
BSA	UCNP@BSA-RB&IR825	Imaging-guided combined photothermal and photodynamic therapy	The theranostic agent irradiated with dual lasers at 808 nm and 980 nm show stronger anti-cancer effect than that at individual wavelength both *in vitro* and *in vivo*.	Chen et al., 2014b
BSA	Fe_3_O_4_-BSA@DOX-PEG	Combined MRI diagnostics and chemotherapy	The theranostic agent showed superparamagnetic property and high *T*_2_-relaxivity value, and displayed similar cytotoxicity against HEK293 and C6 cells as the DOX alone.	Semkina et al., 2015
BSA	Gemcitabine-loaded magnetic BSA nanospheres modified with cetuximab	Simultaneous targeting, MRI diagnostics, and double-targeted thermochemotherapy of pancreatic cancer cells	The theranostic agent not only can effectively distinguish different EGFR-expressing pancreatic cancer cells, but also can evaluate non-invasive methods for different targeting effects by MRI. Combined antibodies and magnetic targeting, the theranostic agent can efficiently inhibit or kill AsPC-1 cells.	Wang et al., 2015
BSA	Fe_5_C_2_-BSA-DOX NPs	Multi-stimuli-regulated photo-chemothermal cancer therapy	The NPs could be used for MRI and fluorescence tumor imaging in SK-OV-3 tumor-bearing mice via i.v. injection. Under the synergistic effect of magnetic targeting, PTT and the increased drug release, the NPs have no systemic toxicity and show good SK-OV-3 tumor elimination.	Yu et al., 2016
BSA	Gd:CuS@BSA NPs	PA/MR bimodal imaging-guided tumor-targeted PTT	The NPs have significant SK-OV-3 tumor-targeted PA/MR imaging performance, as well as effective SK-OV-3 tumor ablation.	Yang et al., 2016
BSA	PEG-BSA-imidazole modified with either Cy5.5 or BHQ-3	pH-activatable on/off tumor targeting probe for the theranostic	The theranostic agent displayed significant cytotoxicity for MCF-7 and A549 cells, and showed a strong fluorescence signal in the endosomal region of MCF-7 cells.	Lee et al., 2016
BSA	[FITC]-BSA-Gd/1,3-bis(2-chloroethyl)-1-nitrosourea NPs	MR and fluorescence imaging-guided chemotherapy	The NPs enable dual imaging for real-time tracking of chemotherapeutic agent *in vitro* and *in vivo*, and can also effectively inhibit MBR 261-2 tumor growth.	Wei et al., 2016
BSA	Folate(FA)-BSA-c-PheoA conjugate:GO complex incorporated free PheoA	Active-targeted and pH-responsive theranostic agent for fluorescence imaging-guided PTT and PDT	The theranostic agent showed the strongest fluorescence signal at the MCF7 tumor at 3 h post-injection. After i.v. injection of the theranostic agent followed by 671 nm laser irradiation, the B16F10 tumor was suppressed, and no acute toxicity was observed.	Battogtokh and Ko, 2016
BSA	Prussian blue-BSA-ICG NPs	MR and NIR fluorescence bimodal imaging guided laser mediated combinatorial phototherapy	After i.v. injection of the NPs, time dependent NIR fluorescence signal and MRI signal was increase at the SCC7 tumor site. Upon irradiation of 808 nm laser irradiation, the SCC7 tumor growth was efficiently suppressed without tumor recurrence.	Sahu et al., 2016
BSA	DOX-loaded UCN/ZnPc@FA-BSA-PCL	Simultaneous tumor cell imaging, PDT and chemotherapy	After 4 h of incubation, DOX and UCN fluorescent signals can be clearly detected in HeLa cells. Compared with single PDT or DOX chemotherapy groups, the theranostic agent showed significantly enhanced HeLa cell killing efficiency.	Dong et al., 2016
BSA	BSA-MnO_2_-ICG NPs and BSA-MnO_2_-PTX NPs	MR imaging-guided PTT and MR imaging-guided chemotherapy	Both NPs showed admirable renal and tumor imaging ability as well as significant 4T1 tumor inhibition via i.v. injection.	Pan et al., 2017
BSA	MoS_2_-Gd-BSA	Dual-modality MR and PA imaging-guided PTT	The enhanced MR/PA signals were detected in the 4T1 tumor site post-injection of the theranostic agent. After i.v. injection of the theranostic agent followed by 808 nm laser irradiation, the 4T1 tumor was suppressed, and the negligible toxicity was observed.	Chen et al., 2017a
BSA	Mn_3_O_4_-BSA-EDTA	Multifunctional imaging-guided PTT	The theranostic agent exhibited applicability a *T*_1_-*T*_2_ dual-model MR imaging and strong NIR (700–1000 nm) imaging *in vitro* and *in vivo*. After i.v. injection of the theranostic agent followed by 785 nm laser irradiation, the HCT116 tumor was suppressed, and the low toxicity was observed.	Liu et al., 2017
BSA	Au-BSA-DOX-FA	pH-sensitive theranostics agent for CT imaging and targeting therapy	The highest CT value in MGC-803 tumor arose at 30 min post-injection. The theranostic agent showed selective antitumor activity effects on the MGC-803 tumor and no side effects on normal organs and tissues.	Huang et al., 2017
BSA	Gd_2_O_3_@BSA conjugating Chlorin e6	MR imaging-guided photo-induced therapy	The theranostic agent can be used for tumor localization and visualization the *in vivo* distribution of Chlorin e6. After i.v. injection of the theranostic agent followed by 660 nm laser irradiation, the 4T1 tumor was suppressed, and no influence on the normal tissues was observed.	Zhou et al., 2017
Ferritin	ZW800-labeled ZnF_16_Pc-ferritin- RGD4C	Fluorescence imaging-guided PDT	The theranostic agent showed admirable liver and U87MG tumor imaging ability, significant U87MG tumor inhibition as well as minimal toxicity to normal tissues via i.v. injection.	Zhen et al., 2013b
Ferritin	ZW800-labeled DOX-ferritin- RGD4C	Fluorescence imaging-guided chemotherapy	After injection of the theranostic agent, the fluorescence signals in the tumor were the strongest and two-fold higher than those in the liver. The theranostic agent showed a longer circulation half-life, significant U87MG tumor inhibition as well low cardiotoxicityl via i.v. injection.	Zhen et al., 2013a
Ferritin	IR820 loaded ferritin nanocage	Fluorescence and PA imaging-guided PTT	In 4T1 tumor-bearing mice, the tumor and its margin normal tissue can be clearly identified via fluorescence (550 nm) imaging from 4 to 24 h post-injection. After i.v. injection of the theranostic agent followed by 808 nm laser irradiation, the 4T1 tumor was suppressed, and no significant body weight loss was observed.	Huang et al., 2014
Ferritin	Gd-HPDO3A-apoferritin-curcumin	MR imaging-guided targeting chemotherapy	The theranostic agent induced MR contrast is stronger in MCF-7 cells than in MDA-MB-231cells. The theranostic agent have a significant reduction of MCF-7 cell proliferation at a concentration of 97 μg/ml.	Geninatti Crich et al., 2015
Ferritin	Gd-HPDO3A-L-ferritin-curcumin	MR imaging-guided targeting chemotherapy	Although the theranostic agent showed relatively low MRI sensitivity, it can effectively destroy the viability and self-renewal of MDA-MB-231 and TUBO cells spheres *in vitro*, and to induce the regression of TUBO tumor in mice.	Conti et al., 2016
Ferritin	CuS-ferritin	PET and PA imaging-guided PTT	After i.v. injection, the theranostic agent showed admirable U87MG tumor imaging ability, significant U87MG tumor inhibition as well as low toxic side effects.	Wang et al., 2016b
Ferritin	L-ferritin decorated PLGA for the delivery of paclitaxel and Gd based MRI agent	MR imaging-guided targeting chemotherapy	The theranostic agent can generate sufficient MRI contrast and an increased cytotoxicity against the SCARA5 receptors over-expressed cancer cells.	Turino et al., 2017
Gelatin	Gelatin-iron oxide core/CaP-DOX NPs	pH-responsive theranostics agent for MR imaging-guided chemotherapy	The NPs showed efficient MR contrast and efficient cell uptake toward HeLa cells.	Li et al., 2013
Gelatin	Fe_3_O_4_@gelatin conjugating FITC and Pt(IV) prodrug	Enzyme-stimulated theranostics agent for chemotherapy, MR imaging and fluorescence sensor	The IC_50_ value of the theranostic agent is much lower than free Pt(IV) prodrug. Significant enhancement in MR signals was observed at the tumor site after *in situ* injection of the theranostic agent.	Cheng et al., 2014
Gelatin	Angio-DOX-dendrigraft poly-lysine-gelatin	Simultaneous cancer-targeted fluorescent imaging and chemotherapy	The theranostic agent showed good targeting efficiency, well penetration ability as well as significantly inhibited 4T1 tumor growth.	Hu et al., 2015
Gelatin	DOX-AuNPs@gelatin	Chemotherapy and intracellular imaging	DOX-based fluorescence allows real-time monitoring of drug uptake, release and distribution in MCF-7 cells. Free DOX is more toxic to MCF-7 cells than DOX-AuNPs@gelatin.	Suarasan et al., 2016
Gelatin	DOX- gelatin-EGCG AuNPs	Enzyme-responsive theranostics agent for real-time monitoring and chemotherapy	The theranostic agent could be effectively tracked by monitoring the recovery of the DOX fluorescence signal and shown significantly inhibit the growth of PC-3 cells.	Tsai et al., 2016
Gelatin	Paclitaxel-loaded gelatin oleic acid superparamagnetic NPs	MR imaging-guided chemotherapy	The NPs can be used as *T*_2_-weighted MRI contrast agents in cancer cells. After the tail vein injection, the NPs have longer systemic circulation time and better anticancer activity than Taxol®.	Tran et al., 2017a,b
Transferrin	DOX-Graphene-SiO_2_-coated quantum dots-Tf conjugates	Simultaneous cancer-targeted fluorescent imaging, monitoring and chemotherapy	DOX-based fluorescence allows real-time monitoring of drug release and distribution in HeLa cells. However, the conjugates showed lower toxicity effect than DOX alone on HeLa and HEK293 cell lines.	Chen et al., 2013
Transferrin	PEGylated fluorescent nanodiamond-Tf-DOX	Simultaneous cancer-targeted fluorescent imaging and chemotherapy	It can discriminate L-02 normal cells form HepG2 tumor cells in terms of fluorescence intensity and cytotoxicity.	Wang et al., 2014a
Transferrin	Docetaxel- and ultra bright gold clusters-loaded Tf-TPGS	Simultaneous cancer-targeted imaging and chemotherapy	The theranostic agent showed 71.73 times more potency than Taxotere® after 24 h treatment with MDA-MB-231-luc breast cancer cells. 24 h after the 4th injection on 24th day the fluorescence intensity was not significantly decreased in the tumor, liver and bladder. After i.v. injection of the theranostic agent, the MDA-MB-231-luc tumor was suppressed, and no significant body weight loss was observed.	Muthu et al., 2015
Transferrin	Paclitaxel-loaded Tf-Fe_3_O_4_/mesoporous silica (core/shell)-Cy7 NPs	Simultaneous cancer-targeted NIR fluorescence/MR imaging and chemotherapy	1 to 24 h after injection, the tumor can be clearly visualized. The NPs showed higher anti-cancer activity on HeLa cells than free PTX.	Jiao et al., 2015
Transferrin	Cy5.5-loaded N-NE3TA-Tf	Targeted iron chelation cancer therapy and NIR fluorescence imaging	The theranostic agent displayed significant inhibitory activity against HeLa, HT29 and PC3 cells, and the NIR fluorescence signals of the theranostic agent can be clearly detected in HeLa, HT29, and PC3 cells.	Kang et al., 2016
Transferrin	Docetaxel- and quantum dots-loaded TPGS-Tf	Brain-targeted imaging and chemotherapy	The theranostic agent can effectively cross the blood-brain barrier and show fluorescence in the brain of rats.	Sonali Singh et al., 2016
Transferrin	Tf-IR780 NPs	NIR imaging and PDT/PTT for Tfr-overexpressed tumors	The CT26 tumor and liver can be clearly visualized at 2 h post-injection, while only the CT26 tumor can be clearly visualized at 12 h post-injection. After injection of the NPs followed by 808 nm laser irradiation, the CT26 tumor was effectively suppressed, and no significant adverse effect was observed.	Wang et al., 2016a
Transferrin	Iron-dependent artesunate-loaded Tf-hollow mesoporous CuS NPs	PA imaging and chemo-phototherapy for Tfr-overexpressed tumors	The NPs can be effectively used for tumor imaging, and peritumoral injection is more conducive to tumor imaging than i.v. injection. After peritumoral injection of the NPs followed by 808 nm laser irradiation, the tumor was effectively suppressed.	Hou et al., 2017
Transferrin	Holo-Tf-ICG	Fluorescence and PA dual-modal imaging and PTT for glioma	The theranostic agent can provide high spatial resolution fluorescence and PA imaging for visualization of the distribution of ICG in subcutaneous- and orthotopic- brain tumors. After i.v. injection of the theranostic agent followed by 808 nm laser irradiation, the U87 tumor was effectively suppressed, and no significant adverse effect was observed.	Zhu et al., 2017
Transferrin	Iron oxide NPs conjugating Tf, TAT peptide and Cy7	Simultaneous cancer cell nuclear targeting, NIR/MR imaging and synchronous PTT	The theranostic agent can be used for the A549 tumor imaging, and the best imaging effect is at about 8 h postinjection. After i.v. injection of the theranostic agent, the A549 tumor was effectively eliminated, and no significant adverse effect was observed.	Peng et al., 2017
Transferrin	Protoporphyrin IX-loaded UCNP@Tf NPs	NIR light induced PDT of cancer cells and luminescence bioimaging	Under 980 nm laser irradiation, the NPs can not only kill MDA-MB-231 cells by PDT, but also show clear bright green in MDA-MB-231 cells.	Wang et al., 2017a
Silk	NIR-797-labeled anti-EGFR-iRGD-PTX-silk fibroin NPs	Fluorescence imaging-guided chemotherapy	In HeLa tumor-bearing mice, the NPs can be used for the tumor and liver fluorescence imaging, and the maximum fluorescence intensity in tumor and liver tissues arose at 24 h post-injection. After tail vein injection of the NPs, the HeLa tumor was eliminated, and no significant adverse effect was observed.	Bian et al., 2016
Silk	Nanodiamonds-silk fibroin-DOX	Fluorescence tracking and chemotherapy	–	Khalid et al., 2016
Silk	DOX-loaded sericin/dextran composite hydrogel	Drug monitoring and chemotherapy	After subcutaneous injection, the photoluminescence of hydrogel is long-term stable in C57BL/6 mice without being quenched. After injection the hydrogel into the vicinity of the B16-F10 tumor, the tumor was effectively suppressed, and no significant differences in body weight were observed.	Liu et al., 2016
Zein	Cy5-labeled hydroxycamptothecin @AuNPs-Zein-folate-conjugated polydopamine	Active targeting in drug delivery and cell imaging	The increase of fluorescence signals from the KB tumors was accompanied by the sharp decline in normal tissues at 3 h post injection, and the fluorescence signals at the tumor remains basically constant within 24 h. After i.v. injection of the theranostic agent, the KB tumor was effectively eliminated, and no acute toxicity was observed.	Wang et al., 2017b
Lipoprotein	Boron/Gd agent lipoprotein adducts	MRI/Boron Neutron Capture Therapy	After i.v. injection of the adducts, high MRI signal intensity was observed in the liver and in the tumor region. 30-40 days after neutron irradiation, the tumor growth of mice was negligible.	Alberti et al., 2015
Lactoferrin	Zinc-doped Fe_3_O_4_-saturated bovine lactoferrin	Real-time cancer imaging and simultaneous cancer-targeted therapy	Orally fed the theranostic agent gave a bright dark (*T*_2_) contrast at the Caco2 tumor site. Oral administration of the theranostic agent exhibited significant antitumor efficacy and a nontoxic and biocompatible nature in the human breast cancer and xenograft colon tumors.	Kamalapuram et al., 2016
Lactoferrin	Fe_3_O_4_ -saturated bovine lactoferrin	Real-time imaging and monitoring the effect of drugs in real time	Oral administration of the theranostic agent exhibited significant antitumor efficacy in the human breast cancer tumor.	Kanwar et al., 2016

## Albumin-based nanoformulations

### Albumin and its properties

Albumins are obtained commercially in considerable amount from human serum (human serum albumin, HSA), bovine serum (bovine serum albumin, BSA), rat serum (RSA) and egg white (ovalbumin) (Karimi et al., 2016). Based on practicality, this review focus on HSA and BSA.

BSA shares ca. 76% sequence homology with HSA (Anand and Mukherjee, 2013), and is very soluble with a 69,323 Da molecular weight, consisting of 583 amino acid residues. BSA has an isoelectric point (pI) of 4.7 with a net charge of −18 mV (Anand and Mukherjee, 2013). BSA molecule is heart-shaped, consisting of three repeating domains (labeled I–III), with each of which falling into two sub-domains A and B (Majorek et al., 2012). BSA is extensively adopted for cancer theranostics by virtue of low cost, abundance, ease of purification and proper delivering properties.

HSA, the most abundant plasma protein (35–50 mg/mL human serum), is synthesized in the liver. HSA is a single-chain and non-glycosylated polypeptide with 66,500 Da in molecular weight, consisting of 585 amino acid residues, and heart-shaped with 80 × 80 × 30 Å in ca. dimensions (Sugio et al., 1999). From X-ray crystallographic analyses, the vital difference between BSA and HSA is that the former contains two tryptophan amino acid residues (Trp-135 and Trp-212), whereas the latter has merely one, Trp-214. HSA is very soluble, being extremely robust toward temperature (available at 60°C for 10 h), pH (stable in pH 4–9) and organic solvents. Besides, HSA protein has preferentially been uptaken to tumor interstitium via the pathway of SPARC glycoprotein and gp60 glycoprotein transcytosis. These properties as well as its deficient toxicity, immunogenicity, and biodegradability make it an ideal candidate for cancer theranostics.

### HSA for cancer theranostics

HSA has broadly served as a natural carrier to isolate organic molecules or inorganic oxide, inclusive of IR780, superparamagnetic iron oxide, IR825, and chlorin e6 (Ce6), for synthesizing effective theranostic agents.

#### Individual HSA-dye complexes

**Figure 2:**
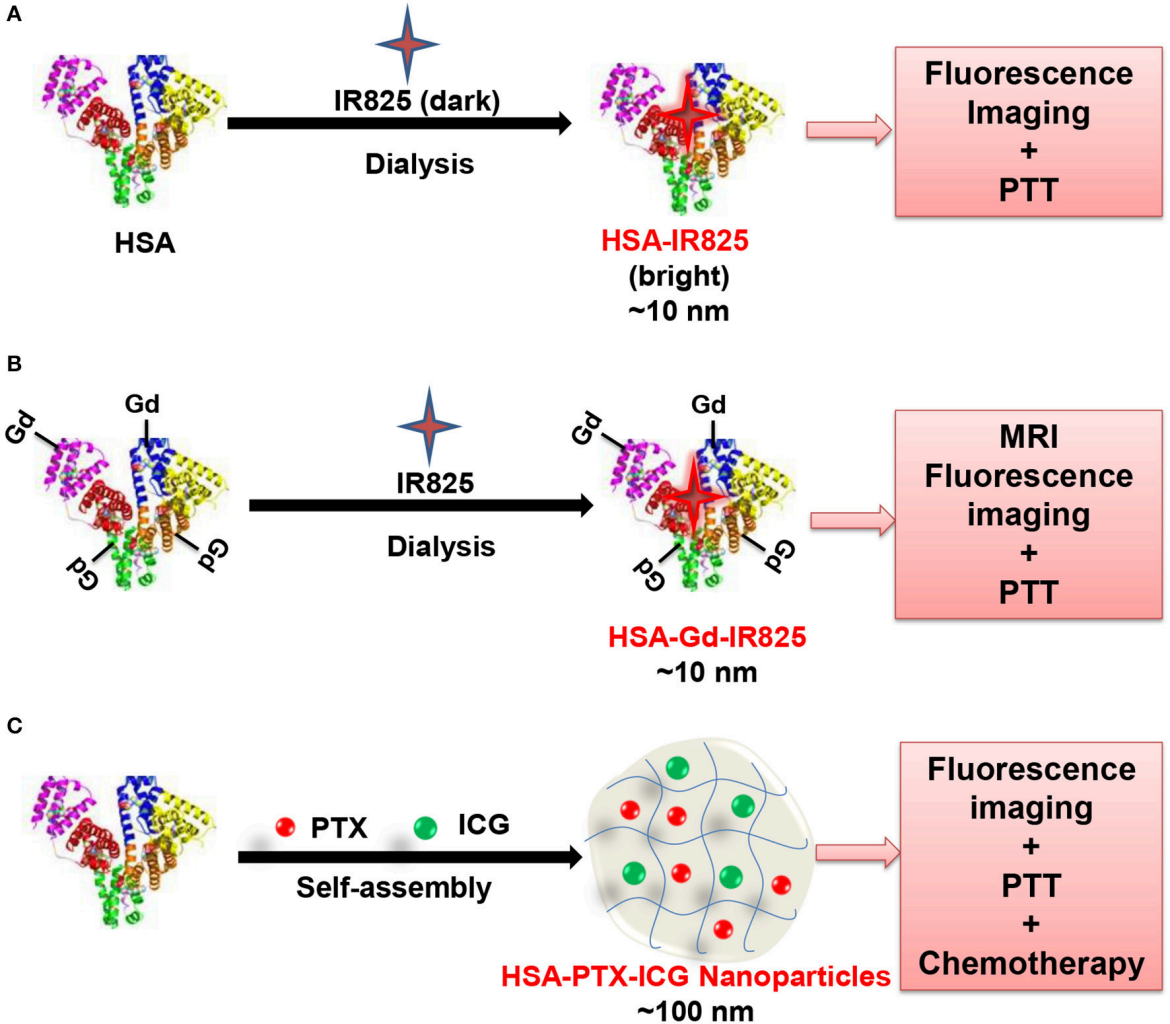
Schematic illustration of the fabrication of HAS-IR825 complex **(A)** (Chen et al., 2014c), HAS-Gd-IR825 complex **(B)** (Chen et al., 2014a), and HSA-ICG-PTX nanoparticles **(C)** (Chen et al., 2015a).

HSA is well-known with multiple hydrophobic binding pockets, and able to be bound non-covalently with many organic dyes, forming HSA-dye complexes with a high fluorescence quantum yield. In recent years, near-infrared (NIR) dyes, e.g., IR780, indocyanine green (ICG) and IR825, are broadly employed for cancer theranostics because of their relatively deep penetration and low interference. IR825 can be bound to the hydrophobic domain of HSA (the molar ratio of 1:1) via hydrophobic interactions, which fabricates a HSA-IR825 complex (Figure 2A) (Chen et al., 2014c). The HSA-IR825 complex have a high fluorescence quantum yield at 600 nm excitation and an even high absorbance whereas low fluorescence quantum yield under 808 nm excitation, showing a great performance in NIR imaging and PTT at separated wavelengths. A gadolinium was further fabricated on HSA-IR825 for dual-modal imaging-guided PTT of tumor in a follow-up study (Figure 2B) (Chen et al., 2014a). In this work, HSA was conjugated with the Gd(III) compound of diethylenetriamine pentaacetic acid, and further complexed with IR825, to form HAS-Gd-IR825 complex. The HAS-Gd-IR825 complex has great fluorescence and NIR absorbance, impressive *T*_1_ relaxivity of 4.82 mM^−1^ s^−1^. More recently, a gemcitabine functionalized HSA-IR780 agent was reported for chemotherapy and imaging-guided PTT of tumor (Han et al., 2017). HSA was first conjugated with gemcitabine via cathepsin B cleavable peptide GFLG, and then mixed with IR780 dye at the molar ratio of 1:1. IR780 could bound to HSA via hydrophobic interactions.

For above systems, the NIR dyes were bound to the HSA via non-covalent interactions, possibly causing dye leakage during *in vivo* circulation (Rong et al., 2015). To address this problem, heptamethine CySCOOH dye (a NIR cyanine dye) was covalently conjugated to the lysine residues of HSA (Rong et al., 2015) via a modified EDC/NHS reaction for effective photoacoustic (PA), NIR fluorescence, thermal multimodality imaging and PTT. Such conjugation, compared with free CySCOOH dye under the identical conditions, resulted in higher PTT efficacy, tumor accumulation and longer circulation. Moreover, the maleimide group can be rapidly and selectively bound to the Cys34 residue of HSA via a Michael addition reaction. Recently, the Michael addition reaction was employed to couple fluorescent dye Cy7 to Cys34 position of HAS (Lisitskiy et al., 2017). And meantime, the chemotherapeutic agent pTFT (5-trifluoromethyl-2′-deoxyuridine 5′-monophosphate) was coupled to lysine residues of HSA via a redox and pH dual-sensitive linker. The conjugates could not only serve as an optical and ^19^F MR imaging, but also be applied for delivery of chemotherapeutics.

#### HSA-based complexes

HSA has an effective diameter of 7.2 nm. Yet nanocarriers with sizes of 100-200 nm are well known to tend to accumulate in tumor tissues more efficiently via the enhanced permeability and retention (EPR) effect (Peer et al., 2007). Besides, theranostic agents usually require more functional ingredients to expand the application. Accordingly, great efforts have been devoted to design HSA-based complexes with appropriate sizes and more characteristics, to establish multifunctional HSA-based theranostics. For example, melanin and paclitaxel (PTX)-loaded HSA nanoparticles (HMP-NPs) with size of ca. 192 nm were fabricated with a desolvation-crosslinking method (Mo et al., 2007; Sim et al., 2015). The HMP-NPs showed effective PA signal intensity in the tumor site and the capability to tumor chemotherapy with long circulation time, as confirmed by *in vivo* experiments. In addition, the desolvation-crosslinking method was adopted to form HSA-based NPs (Pt(IV)-probe@HSA) for theranostic application (Li et al., 2015). In this work, the HSA NPs surface were conjugated with Pt(IV) antitumor prodrug, NIR fluorophore Cy5, and quencher Qsy21. The Pt(IV)-probe@HSA can not only selectively trigger the localized activation of Pt(IV) prodrug, but also enable real-time tumor cell imaging with high resolution.

For the above systems, glutaraldehyde is employed to stabilize the HSA NPs, whereas possible aldehyde residue may cause some side effects for *in vivo* applications (Fürst and Banerjee, 2005). To address this problem, some facile methods involving no toxic chemicals or exogenous cross-linkers have been broadly adopted in the preparation (Sheng et al., 2014; Hu et al., 2016). As an example, HSA-ICG NPs was developed with an average hydrodynamic diameter of nearly 75 nm based on the intermolecular disulfide bond cross-linking within HSA, for imaging-guided PDT and PTT treatments (Sheng et al., 2014). In this work, they first broke up the intramolecular disulfide bonds of HSA with the endogenous reducing agent glutathione, and then fabricated the HSA NPs with a desolvation method. A similar strategy was also adopted in their recent work to fabricate HSA nanoassemblies (NAs) with photosensitizer chlorin e6 (HSA-Ce6 NAs) for multi-modal imaging-guided PDT (Hu et al., 2016). The proposed HSA-Ce6 NAs had a diameter of ca. 100 nm, excellent tumor selectivity, promising triple-modal (fluorescence, PA and MR) imaging, and effective PDT properties.

Another method proposed by Liu's group not introducing exogenous cross-linkers is to fabricate HSA-based multifunctional theranostic NPs with drug-induced protein assembly strategy (Chen et al., 2015a). In this work, a multifunctional “Abraxane-like” theranostics agent was formulated through simply incorporating three clinical approved agents, ICG, PTX and HSA together (Figure 2C). In this formulation, the NIR fluorescence imaging, thermal imaging, PTT and chemotherapy were efficiently combined. Accordingly, a synergistic therapeutic effect is demonstrated in treating metastatic and subcutaneous breast tumors. As a result, the strategy is enriched to design tumor-targeted theranostics agent for multimodal imaging-guided therapy of tumors (Chen et al., 2015b). HSA is pre-modified in their design, with either a tumor-targeting acyclic Arg-Gly-Asp (RGD) peptide (HSA-RGD) or a photosensitizing agent Ce6 (HSA-Ce6). The anticancer drug PTX is then employed to induce the self-assembly of HSA-RGD and HSA-Ce6 to fall into two different NPs. After incorporation of manganese(II), both of NPs could be tracked by MR imaging and fluorescence imaging, which can be adopted for combinatorial cancer PDT and chemotherapy. Similarly, photosensitizer agent iron (II) phthalocyanine (FePc)-induced HSA self-assembly, is employed in a recent study to fabricate multifunctional HSA-FePc NPs for PA imaging-guided PTT (Jia et al., 2017). The as-prepared HSA-FePc NPs exhibited high stability, high PA imaging quality, efficient PTT treatment, and low long-term toxicity *in vivo*.

#### HSA-coated complexes

PTX-loaded HSA NPs were conjugated to the surface of porphyrin microbubbles for cancer theranostics (Moon et al., 2015). In this system, porphyrin microbubbles were fabricated using porphyrin-phospholipid conjugates to simultaneously intensify ultrasound and PA signal. The developed multifunctional theranostics agent is high sensitive in PA and ultrasound imaging, and effective in delivery of anticancer drug PTX to a tumor site. HSA-coated superparamagnetic iron oxide NPs were presented more recently, which can absorb pulsed microwave energy and transform efficiency into shockwave with the thermoelastic effect besides using as MR contrast agents (Wen et al., 2017).

### BSA for cancer theranostics

#### Individual BSA–dye complexes

BSA consists of multiple hydrophobic binding sites, and can naturally serve as a carrier of numerous small NIR dyes agents. Squaraine (SQ) was selectively bound to hydrophobic domain of BSA via hydrogen bonding and hydrophobic interactions with 80-fold enhanced fluorescence intensity (Gao et al., 2014). Based on this, a supramolecular adducts of SQ and BSA (SQ@BSA) was constructed and served as PTT agent and effective bioimaging probe simultaneously. In addition, folic acid (FA) functionalized SQ@BSA (SQ@BSA-FA) has additional functions, e.g., monitoring the time-dependent bio-distribution of adducts and targeting tumor sites. ICG, an FDA-approved NIR dye, can be adsorbed on BSA for NIR fluorescence imaging, PTT and PDT as excited by single-wavelength (Chen and Liu, 2016). Besides, BSA-based theranostics system covalently modified with NIR dyes has been reported (Lee et al., 2016). For example, a zinc-coordinated pH-sensitive theranostics agent is reported, consisting of two types of polyethylene glycol-BSA-imidazole covalently modified with either BHQ-3 quencher (NIR dark quencher) or Cy5.5 dye (donor NIR dye). At pH 5.0 (e.g., endo/lysosomes in cancer cells), the theranostics agent was disassembled rapidly, and emitted strong NIR fluorescence (Lee et al., 2016).

#### BSA-based complexes

**Figure 3:**
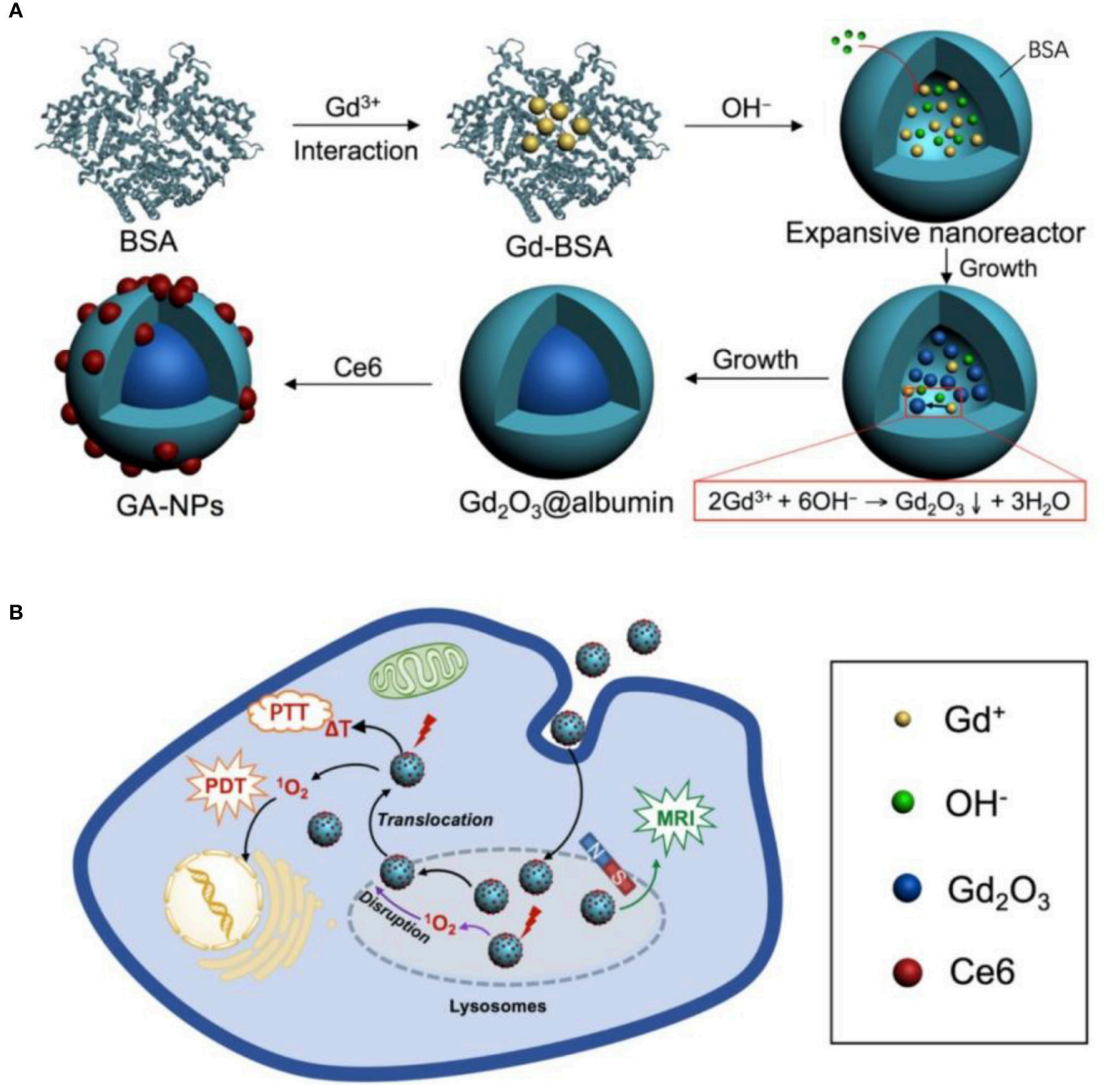
Schematic illustration the synthesis of core-shell Gd_2_O_3_@BSA conjugating Ce6 **(A)** for MRI-guided cancer photothermal therapy (PTT) and photodynamic therapy (PDT) **(B)**. Reproduced with permission (Zhou et al., 2017).

Besides bound to NIR dyes, BSA can be further engineered with other functional agents via complexation, such as gadolinium (Wei et al., 2016; Yang et al., 2016; Chen et al., 2017a; Zhou et al., 2017), manganese (Liu et al., 2017; Pan et al., 2017) and graphene's derivatives (Sheng et al., 2013). For example, hollow BSA was employed to fabricate a size-tunable Gd_2_O_3_@BSA conjugating Ce6 theranostics agent for MR imaging-guided PDT and PTT (Figure 3) (Zhou et al., 2017). Notably, the BSA nanoreactor can not only effectively regulate the longitudinal relaxivity of Gd_2_O_3_, but conjugate readily with photosensitizers. A facile strategy is presented, adopting BSA as a biotemplate at physiological temperature, to construct a biocompatible Gd-integrated CuS multifunctional theranostics agent (Gd:CuS@BSA) (Yang et al., 2016). The fabricated Gd:CuS@BSA theranostics agent possessed ultrasmall sizes (about 9 nm), acceptable longitudinal *r*_1_ relaxivity of 16.032 mM^−1^·s^−1^, impressive temperature rise and intense PA signals under NIR irradiation. Recently, a multifunctional MoS_2_-Gd-BSA theranostics agent was fabricated through incorporating the good photothermal effect of MoS_2_ nanoflakes with the high longitudinal proton relaxivity of BSA-Gd complex via the amine reaction between carboxyl groups of MoS_2_ nanoflakes and amino groups of BSA-Gd (Chen et al., 2017a). The MoS_2_-Gd-BSA theranostics agent exhibits a strong NIR absorbance and high *r*_1_ relaxivity, which are helpful for PA and *T*_1_-weighted MR dual-modal imaging-guided PTT of cancer. Besides, a chemotherapeutic drug carmustine-encapsulated FITC-BSA NPs were prepared by desolvation/denaturation method and conjugated with a MR gadolinium(III) salt to form nanomedicine with dual imaging modalities (Wei et al., 2016).

At the identical stage, contrast agent Mn-based BSA complexes have been also reported for cancer theranostics (Liu et al., 2017; Pan et al., 2017). For example, a multifunctional BSA-MnO_2_ theranostics agent was fabricated via a mimicking drug-substrate interaction strategy, adopting BSA as both reductant agent and template (Pan et al., 2017). It is noteworthy that ICG and PTX can be loaded on BSA-MnO_2_ with a facile and energy-saving mixing. The ICG/PTX-loaded BSA-MnO_2_ theranostics agent could be adopted for MR imaging guided PTT and chemotherapy *in vitro* and *in vivo*.

Furthermore, a BSA-assisted synthesis route was developed to produce reduced graphene oxide (nano-rGO) theranostic agent (Sheng et al., 2013). The BSA functionalized nano-rGO displayed a high stability and low cytotoxicity, enabling PA imaging and PTT treatment without further surface modification. On that basis, a folate receptor active-targeted, pH-responsive BSA-pheophorbide-a (PheoA) photosensitizer incorporated GO nanocarrier (PheoA + GO:FA-BSA-c-PheoA NC) is developed as an theranostic agent (Battogtokh and Ko, 2016). The theranostic agent carries a good pH-responsive photosensitizer and generates a synergistic PTT and PDT effect against tumor by NIR irradiation.

#### BSA-coated complexes

BSA serves not only as a delivery platform to load various functional molecules, e.g., dyes and chemotherapy drugs, but also as a coating agent of other nanocarriers to increase the physiological stability, water-solubility and blood circulation time of theranostics systems. For example, BSA is employed to coat NaGdY_4_-based upconversion NPs (UCNPs), resulting in UCNP@BSA NPs possessing well stability and water-solubility in physiological environments (Chen et al., 2014b). And meantime, two different dyes, consisting of an IR825 dye and a Rose Bengal (RB) photosensitizer, can be effectively loaded onto the BSA shell layer of the UCNP@BSA system. It is noteworthy that the characteristic absorbance peak of RB complies well with the green emission peak of UCNPs (980 nm excitation), which effectively kills cancer cells by PDT. Therefore, the dual-dye loaded theranostics agent can be adopted for MR diagnostic, upconversion optical imaging, PDT as well as PTT both *in vitro* and *in vivo*. BSA-coated magnetite Fe_3_O_4_ core-shell structures with anticancer drug (gemcitabine) were developed, where BSA serving as the outer shell was further functionalized by the active targeted agent Anti-EGFR mab C225 (Wang et al., 2015). The theranostics agent can efficiently regulate double-targeted thermochemotherapy against pancreatic tumor, monitor different cellular targeting by MR imaging, and distinguish various EGFR-expressing pancreatic tumor cells. On that basis, a BSA-coated magnetite Fe_5_C_2_ theranostics agent was developed with a high loading of antitumor drug doxorubicin (DOX) (Yu et al., 2016). Notably, the DOX can be released in acidic condition and irradiated by NIR. In this regard, the theranostics agent serves as a smart nanoplatform for MR imaging, effective chemotherapy and PTT. A pH-responsive protein–polymer bioconjugate-coated theranostics agent consisting of a superparamagnetic magnetite Fe_3_O_4_ core, BSA–poly(ethylene glycol) (PEG) shell and anticancer drug DOX were presented for combined MR imaging diagnostics and chemotherapy (Semkina et al., 2015). A BSA–poly(ε-caprolactone) bioconjugate-coated upconversion theranostics agent is constructed with the similar preparation method for simultaneous cancer cell imaging, PDT, and chemotherapy (Dong et al., 2016). BSA stabilized Prussian blue (PB) NPs were fabricated, and ICG molecules were further attached non-covalently by a biocompatible and simple method (Sahu et al., 2016). Here, PB serves as a MR contrast enhancer. Accordingly, the multifunctional theranostics system could provide dual mode MR signal and NIR fluorescence imaging as well as combined therapy with PDT and PTT. A pH-responsive Au–BSA core/shell theranostics agent consisting of Au core, BSA shell that conjugated with DOX and FA was developed recently, which manifested tumor computed tomography (CT) imaging application and targeted cancer therapy (Huang et al., 2017).

Albumin-based NPs could be synthesized by using albumin as a scaffold, template, or stabilizer, and conjugating to polymers, drugs, and contrast agents. Covalent and non-covalent conjugation or assembly were employed. Due to the success of Abraxane, researchers preferred the non-convalent self-assembly. However, NPs prepared using the non-convalent strategy will vary batch to batch, which need to be overcome in the future studies (An and Zhang, 2017).

## Ferritin-based nanoformulations

### Ferritin and its properties

Ferritin counts as an abundant protein in circulation, existing in intracellular and extracellular compartments. Ferritin is a 450 kDa hollow nanocage with internal and external dimensions of 8 and 12 nm, respectively (Banyard et al., 1978). It accumulates and stores approximately 4,500 iron atoms in a non-toxic whereas bioavailable form (Alkhateeb and Connor, 2013). In mammals, each ferritin protein consists of 24 subunits self-assembled into a spherical symmetrical protein shell (Alkhateeb and Connor, 2013). The ferritins from eukaryotes are produced by self-assembly of two subunit types, i.e., L-ferritin chain (19 kDa) and H-ferritin chain (21 kDa). The H-chain is centered by an iron oxidase center, required to oxidize Fe(II) to Fe(III), whereas the L-chain without ferroxidase activity nucleates iron (Bellini et al., 2014). Ferritin counts as a multifunctional protein with iron storage and metabolism. Ferritin is critical for angiogenesis, proliferation and immunosuppression, as demonstrated by growing number of evidence (Alkhateeb and Connor, 2013).

Ferritin is a ubiquitous protein robust extremely: it can be reversibly disassembled in the extremely acidic pH (pH 2–3) or basic pH (pH 10–12) and can be heated at 80°C exerting no deleterious effects (Truffi et al., 2016). These properties as well as its biocompatibility and biodegradability make it a suitable candidate for cancer theranostics (Bhushan et al., 2014).

### Ferritin for cancer theranostics

The ferritin not only provides a reaction vessel to fabricate numerous non-native metallic NPs inside its core, but also serves as a nanocarrier for various applications (Bhushan et al., 2014). For example, Zn hexadecafluorophthalocyanine (ZnF_16_Pc), a potent hydrophobic photosensitizer, is well encapsulated into the Cys-Asp-Cys-Arg-Gly-Asp-Cys-Phe-Cys (RGD4C)-modified ferritins (P-RFRTs) with a loading rate as high as ~60 wt % for effective PDT (Zhen et al., 2013b). The P-RFRTs were further conjugated with ZW800 (a NIR dye molecule) in this work to better track P-RFRTs particles. With the identical approach, the DOX-loaded RGD4C-modified ferritin was also reported (Zhen et al., 2013a). A self-assembly encapsulation strategy based on step-wise change of pH was developed in another report to fabricate ferritin NPs with NIR dye IR820 for fluorescence/PA multimodal imaging-guided PTT (Huang et al., 2014). The IR820-loaded ferritin nanocages can effectively treat and diagnose cancer adopting two different excitation wavelengths, i.e., 550 nm for high quantum-yield fluorescence imaging, and 808 nm for PA imaging and effective PTT. Additionally, CuS was fabricated inside the cavity of ferritin nanocages with a biomimetic and straightforward synthesis strategy (Wang et al., 2016b). The CuS-ferritin has strong NIR absorbance, high photothermal conversion efficiency, good biocompatibility and distinct PA contrast,. Notably, the ^64^CuS-ferritin theranostic system, as incorporated with radionuclide ^64^Cu, also served as an excellent PET imaging agent.

The higher level of L-ferritin in tumor versus normal tissue has been observed in some malignancies tissues like breast cancer, colon cancer, pancreatic cancer and testicular seminoma (Alkhateeb and Connor, 2013). High amount of L-ferritin is usually associated and bound with intensified expression of the L-ferritin receptor that mediates ferritin endocytosis. The higher the expression of L-ferritin receptors, the more intensified uptake of L-ferritin in breast cancer MCF-7 cells (Geninatti Crich et al., 2015). Based on this, a ferritin-based nanotheranostic system has been further developed to simultaneously deliver a MR contrast agent GdHPDO3A and a natural anticancer molecule curcumin (Geninatti Crich et al., 2015). The theranostics system selectively delivered therapeutic and imaging agents to breast cancer cells. In a follow-up study, L-ferritin was found to target breast cancer stem cells (Conti et al., 2016). In this regard, ferritin was further exploited to deliver curcumin and the GdHPDO3A MR contrast agent simultaneously for breast cancer stem cells (Conti et al., 2016). In a very recent report (Turino et al., 2017), multifunctional theranostics system was developed through coating poly (lactic-co-glycolic acid) NPs (PLGA NPs) with L-ferritin to increase their targeting capability to breast cancer MCF-7 cells. The L-ferritin functionalized PLGA NPs loaded with an amphiphilic Gd based MR contrast agent and PTX for MR imaging guided chemotherapy. In addition to this, ferritin coating makes PLGA NPs more stable, thus avoiding the non-specific and fast release of therapy/diagnosis agents before reaching the targeted sites.

Hollow structure make the ferritin become an ideal carrier, however, after loading therapeutic agents, the slight change in conformation and activity might take place. The toxicity of ferritin-based NPs should be studied carefully as other NPs. Also, the surface-modified ferritins may be recognized as foreign substances. Therefore, when surface targeting ligands were needed, the grafting density are important factors to be considered.

## Gelatin-based nanoformulations

### Gelatin and its properties

Gelatin is a polyampholyte protein having both anion and cation along with hydrophobic groups (Elzoghby, 2013). Gelatins have repeating sequences of proline, alanine, and glycine amino acid triplets, which are essential for gelatin (Sahoo et al., 2015). They are obtained from alkaline-, acid- or enzymatic- hydrolysis of collagen. These chemically treated gelatins have varying isoelectric points, i.e., 4.5–6.0 for alkaline treatment and 7–9 for acid treatment (Patel et al., 2008; Ninan et al., 2011). In addition, anticancer agent release profiles from gelatin protein can be controlled through changing the molecular weight, gelatin source and the crosslinking degree (Foox and Zilberman, 2015). Gelatin is natural, biocompatible, biodegradable, water permeable, non-toxic, and soluble in water (Nezhadi et al., 2009). As a delivery carrier of therapeutic/diagnostic agents, gelatin has been revealed multifunctional properties, enabling the development and design of versatile theranostics.

### Gelatin for cancer theranostics

Gelatin has native hydrophobic and hydrophilic segments in each single polypeptide chain, enabling itself a conducive coating agent for various metal NPs (Li et al., 2013; Cheng et al., 2014; Tran et al., 2017b). For example, a theranostic system was developed through coating iron oxide NPs with self-assembled gelatin (abbreviated as AGIO) (Li et al., 2013). In the meantime, calcium phosphate (CaP) and anticancer DOX could be loaded on gelatin by electrolytic co-deposition technique. The fabricated AGIO@CaP-DOX NPs had efficient MR contrast, superior cytocompatibility and potent cellular internalization toward HeLa cells. A gelatin coated multifunctional nanosystem, with oleylamine-coated Fe_3_O_4_ NPs core, amphiphilic gelatin shell, and fluorescent labeling molecules FITC and antitumor platinum(IV) prodrug, was elaborated for fluorescence and MR imaging guided chemotherapy (Cheng et al., 2014). Most recently, oleic acid and gelatin were adopted to modify a silica-coated iron oxide magnetic NPs, which was demonstrated to encrease biocompatibility and solubility of iron oxide magnetic NPs and facilitate treatment-response monitoring of the tumors (Tran et al., 2017b). Particularly, the oleic acid and gelatin coated NPs enables the hydrophobic anticancer drug PTX to be loaded into the lipophilic oleic acid-gelatin shell. The synthesized theranostic system have high r_2_ value, low cellular toxicity, high drug delivery efficacy and well anticancer efficacy *in vitro*. The pharmacokinetics, bio-distribution, tumor diagnostic and antitumor efficacy of the theranostic system *in vivo* were studied in a follow-up study to further confirm its potential in clinical applications (Tran et al., 2017a).

Beside iron oxide NPs, gold NPs are commonly coated with gelatin to increase their stability and biocompatibility. For example, a gelatin-coated gold NP-based nanostructure was developed for fluorescence imaging-guided chemotherapy (Tsai et al., 2016). The gelatin was firstly covalent conjugated with DOX, and then coated onto the epigallocatechin gallate (EGCG)-functionalized Au NPs. Here, DOX serves both as an anticancer drug and a fluorescent indicator. The attained DOX-gelatin/EGCG Au NPs exert an apparent inhibitory effect on the proliferation of human prostate cancer cells (PC-3) and can trace the intracellular enzyme-induced release of DOX through measuring the recovery of the DOX fluorescence signal. The covalent conjugation of DOX to nanocarriers may cause problems, e.g., insufficient *in vivo* release and decreased drug activity (Suarasan et al., 2016). A new temperature- and pH-responsive theranostic system was developed on the basis of DOX non-covalently bound to biosynthesized gelatin-coated Au NPs (DOX-AuNPs@gelatin) (Suarasan et al., 2016). The fabricated DOX-AuNPs@gelatin would be an ideal agent for cancer theranostics based on its good biocompatibility, high DOX loading capacity via non-covalent complexation and effective DOX release under the tumor environment.

Recently, angiopep-2 modified gelatin-based core-shell NPs (Angio-DOX-DGL-Gel-NP) have been designed to increase the tumor targeting efficiency, tumor retention and tumor penetration (Hu et al., 2015). In this system, the shell consisted of dendrigraft poly-lysine linked with angiopep-2 and DOX, whereas the core was made up of gelatin NPs degraded by matrix metalloproteinase-2. Fluorescence imaging-guided chemotherapy showed enhanced antitumor effects *in vitro* and *in vivo*,

Gelatins are used as carriers in versatile drug delivery systems, form nanoparticles to microparticles (Foox and Zilberman, 2015). Small drug molecules or large bioactive molecules were easily entrapped into gelatins and released in a controlled manner. However, the cancer theranostic platforms based on gelatin didn't attract much attention. In our opinion, the gelatin-based NPs are promise cancer theranostics, especially in oral administration and brain delivery.

## Transferrin-based nanoformulations

### Transferrin and its properties

Transferrin is a monomeric glycoprotein with 679 amino acids and a large molecular weight of approximately 79 kDa (Parkkinen et al., 2002; Gomme et al., 2005). The molecule is protected by 3 carbohydrate side chains, one of which is O-linked (Ser-32) and the other are N-linked (Asn-413 and Asn-611) (Gomme et al., 2005). The polypeptide chain falls into two structurally similar lobes, known as the C-lobe (343 amino acids) and N-lobe (336 amino acids), which are connected by a short linear spacer sequence (Brandsma et al., 2011). Each lobe consists of one reversibly binding site for ferric iron with nearly 10^22^ M^−1^ affinity at pH 7.4 (Aisen et al., 1978). The major biological function of transferrin is to distribute and control circulating Fe, which is required for numerous biological processes, including cellular metabolism and proliferation, DNA synthesis, electron transfer, and oxygen transport (Dufès et al., 2013; Tortorella and Karagiannis, 2014). Particularly, transferrin specifically binds transferrin receptors (TfRs) on cell surface, forming a transferrin-TfR complex, and gets internalized by receptor-mediated endocytosis (Szoke and Panteghini, 2012; Dufès et al., 2013). Meantime, overexpression of TfRs has been observed in various cancer cells, including breast carcinoma, glioblastoma, melanoma, ovarian carcinoma and colon carcinoma (Calzolari et al., 2007; Tros de Ilarduya and Düzgüneş, 2013).

### Transferrin for cancer theranostics

As a promising tumor targeting ligand, transferrin is frequently adopted to facilitate targeting delivery of theranostics. For example, transferrin–functionalized graphene quantum dots were developed to track and image tumor cells expressing the TfRs (Chen et al., 2013). In the meantime, the anticancer drug, DOX, was adsorbed on graphene surface in the system via hydrophobic interactions and π-π stacking, which could be used for fluorescence imaging-guided chemotherapy. Another transferrin-conjugated PEGylated fluorescent nanodiamond with Dox payload was developed, and its targeting ability and chemotherapeutic potential were investigated in the human hepatoma (HepG2) cell lines with overexpression of TfRs and normal cell lines (L-02) with low-expression of TfR, respectively (Wang et al., 2014a). A transferrin conjugated theranostic micelles of D-alpha-tocopheryl PEG 1000 succinate (TPGS) were synthesized, containing both ultra-bright gold clusters as a model imaging agent and docetaxel as anticancer drug for synchronous cancer imaging and therapy (Muthu et al., 2015). The transferrin conjugated micelles, compared with the non-transferrin functionalized theranostic micelles, showed higher cellular uptake, higher cytotoxicity in MDA-MB-231-luc breast cancer cells. In a follow-up study, a transferrin decorated TPGS coated theranostic liposomes was further developed for targeted co-delivery of quantum dots and docetaxel for imaging-guided chemotherapy of brain cancer (Sonali Singh et al., 2016). Indeed, the transferrin decorated theranostic liposomes, compared with the non-transferrin targeted preparations, showed an improved and prolonged brain targeting of quantum dots and docetaxel. In addition, rattle-type theranostic NPs of mesoporous silica-coated Fe_3_O_4_ were develop by conjugating transferrin for targeted co-delivery of NIR dye (Cy7) and hydrophobic anticancer drug PTX for NIR/MR bimodal imaging guided chemotherapy (Jiao et al., 2015).

Recently, transferrin and nuclear-targeted TAT peptide (YGRKKRRQRRR) conjugated magnetic NPs were proposed for PTT application (Peng et al., 2017). Magnetic NPs is pre-conjugated with transferrin and TAT peptide, and then bound to NIR dye Cy7. The theranostic system can efficiently target cancer cell nucleus and facilitate the NIR and MR imaging-guided PTT. A core-shell theranostic system UCNP core (NaYF_4_:Gd^3+^, Yb^3+^, Er^3+^) was developed, taking advantage of efficient NIR-to-visible up-conversion capability and increased tumor targeting ability and biocompatibility (Wang et al., 2017a). In the meantime, a clinically approved PTT agent, protoporphyrin IX, was loaded into the transferrin shell, being able to be drawn upon by cancer cells for efficient PDT with NIR irradiation and luminescence bio-imaging. A new strategy (diffusion molecular retention tumor targeting effect) was developed to fabricate tumor-targeted theranostic system for PA imaging-guided chemotherapy/photothermal with synergistic effect (Hou et al., 2017). In this study, hollow mesoporous CuS NPs (HMCuS NPs) were conjugated with transferrin and iron-dependent artesunate (AS), an effective anticancer drug. In this regard, the attained AS/Transferrin-HMCuS NPs facilitated local drug accumulation and retention, targeted to breast cancer MCF-7 cells specially via TfR-mediated endocytosis, combined chemotherapy-phototherapy synergistically, and eventually improved the anticancer effect.

**Figure 4:**
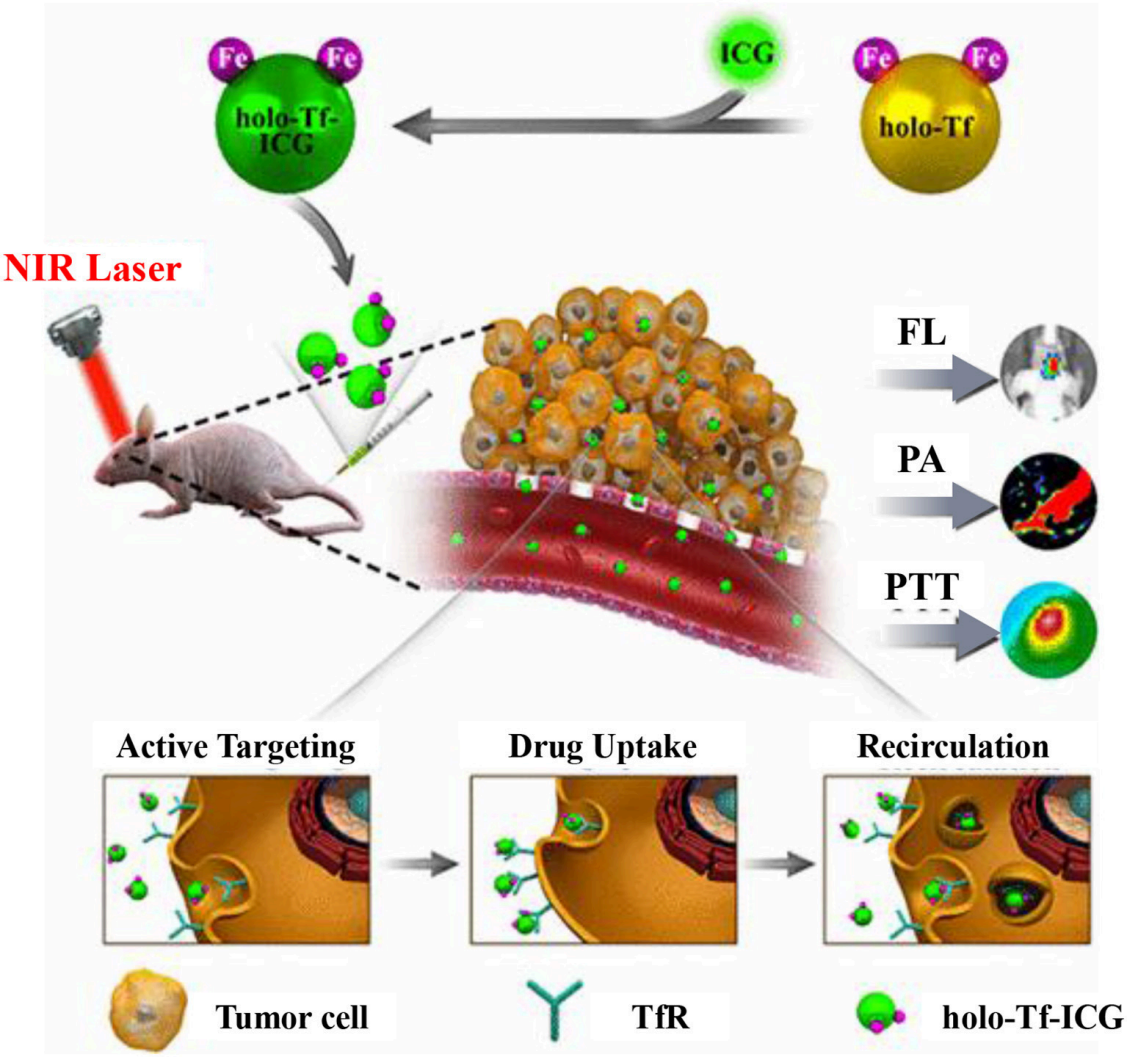
Schematic illustration of holo-Transferrin-ICG complex for dual-modal imaging-guided cancer photothermal treatment *in vivo*. Reproduced with permission (Zhu et al., 2017). Copyright (2017) American Chemical Society.

Transferrin is not only a conducive targeting ligand, but also able to load imaging and/or therapeutic agents. For example, a simple, effective and safe self-assembly strategy was developed to fabricate transferrin NPs with NIR dye IR780 (Transferrin-IR780 NPs) for targeted imaging and phototherapy of cancer (Wang et al., 2016a). The fabricated Transferrin-IR780 NPs had advantages on potent photo-stability, narrow size distribution as well as prominent photothermal conversion efficiency, and exhibited pronounced targeting and theranostics potential. A conjugate of transferrin, NIR dye Cy5.5 and cytotoxic chelating agent (NNE3TA:2,2′-(7-(2-((carboxymethyl)(4-nitrobenzyl)amino)ethyl)-1,4,7-triazonane-1,4-diyl)diacetic acid) (NNE3TA-Transferrin-Cy5.5) was developed to treat and detect cancers (Kang et al., 2016). Targeted iron chelation cancer therapy and NIR imaging were demonstrated effectively *in vitro* with NNE3TA-Transferrin-Cy5.5. Most recently, a drug-induced transferrin self-assembly strategy is developed to fabricate tumor-targeted NPs for fluorescence and PA dual-modal imaging-guided PTT of glioma (Figure 4) (Zhu et al., 2017). In this system, transferrin could effectively load ICG via hydrophobic interaction and hydrogen bonding. Specifically, the preparation method is safe, simple and mild without the use of any toxic reagents. The obtained Transferrin-ICG NAs showed effective active tumor-targeting, good biocompability, prominent dual-modal imaging as well as PTT efficacy, and could be adopted for theranostics of both subcutaneous and orthotopic brain tumors.

Notably, transferrin was employed not only as an delivery vehicle but also as a targeting agent in these works. It has been reported than transferrin-functionalized silica NPs lost targeting abilities completely in serum-rich media, due to shielding effects of the adsorbed proteins (Salvati et al., 2013). The bioavailability of the large transferrin determines the targeting functionality in complex biological media. It is a challenge to maintain the targeting efficiency of transferrin-based NPs *in vivo*.

## Others

With the exception of the foregoing proteins, several other proteins, e.g., silk fibroin (Bian et al., 2016; Khalid et al., 2016; Liu et al., 2016), zein (Wang et al., 2017b), lipoprotein (Mathew et al., 2013; Alberti et al., 2015) and lactoferrin (Kamalapuram et al., 2016; Kanwar et al., 2016), have also been successfully employed for cancer theranostics, as detailed in Table 1. For instance, multimodular zinc-doped Fe_3_O_4_-saturated bovine lactoferrin NPs (Zn-Fe-bLf NPs) were fabricated for a targeted theranostics application through oral administration (Kamalapuram et al., 2016).

Although natural proteins were used in most studies, the disadvantages of these protein-based platforms were obvious, such as poor permeability, ease of degradation, and potential immune responses. Moreover, after functionalization or conjugation of other molecules, the protein properties and functions might be altered. In recent years, encapsulation of proteins by *in situ* polymerization was developed as an alternative strategies for protein modification (Ye et al., 2016b). The protein nanogels were obtained by incorporation of cross-linkers to protein surface and polymerization. Such protein nanogels can maintain the protein properties and functions in complex chemical or biological environment, and avoid the immunogenicity of proteins. The out layer polymers were cleavable and degradable, while the inner proteins could be released by internal and external stimuli. Numerous therapeutic proteins have been developed using this method for cancer therapy (Ye et al., 2016a, 2017).

## Conclusion and prospects

Natural proteins as biocompatible nanocarriers are broadly adopted in delivering therapeutic and diagnostic agents simultaneously. These nanoformulations are fabricated progressively complex and “smart” to employ multi functions in one platform. To increase the accuracy of diagnostic and the efficiency of cancer therapy, “all-in-one” nanoplatforms are designed with more than two imaging strategies and more than two therapeutic methods. Surface modification with targeted molecules and controlled drug release are employed to spatially control the localization of administered NPs. The improved efficiencies in tumor imaging and ablation are verified both *in vitro* and *in vivo*. Despite these huge advances, the translation of natural protein nanoplatforms from laboratory to clinical trials remains an enormous challenge. More efforts should be made to improve the NP reproducibility, colloidal stability in biological environments, and drug-loading efficiency. Furthermore, live and spleen accumulation is a big barrier in clinical translation of all types of NPs. Accordingly, more understanding of bio-nano interactions is required, while more data of pharmacokinetics should be collected and analyzed. Long-term toxicity of nanoformulation is not well evaluated. In addition to mouse model, more animal models, e.g., beagle dogs, monkeys and gorillas, should serve for assessing the long-term toxicity and therapeutic effect. Engineering complex particles with multiple functions are currently achieved by chemists. Accurate diagnose of early stage of tumor and precision drug delivery to tumor site should be more considerable in the future developing of protein-based cancer theranostics.

## Author contributions

YG, HZ, and GS: designed this work of review; DM, MZ, and LW: performed the literature search of the databases; YG: wrote the manuscript; HZ and GS: revised the manuscript. All authors approved the paper for publication.

### Conflict of interest statement

The authors declare that the research was conducted in the absence of any commercial or financial relationships that could be construed as a potential conflict of interest.
